# Chemophototherapy: An Emerging Treatment Option for Solid Tumors

**DOI:** 10.1002/advs.201600106

**Published:** 2016-05-24

**Authors:** Dandan Luo, Kevin A. Carter, Dyego Miranda, Jonathan F. Lovell

**Affiliations:** ^1^ Department of Biomedical EngineeringUniversity at BuffaloState University of New YorkBuffaloNY14260

**Keywords:** chemophototherapy (CPT), chemotherapy, photodynamic therapy, phototherapy, tumor ablation

## Abstract

Near infrared (NIR) light penetrates human tissues with limited depth, thereby providing a method to safely deliver non‐ionizing radiation to well‐defined target tissue volumes. Light‐based therapies including photodynamic therapy (PDT) and laser‐induced thermal therapy have been validated clinically for curative and palliative treatment of solid tumors. However, these monotherapies can suffer from incomplete tumor killing and have not displaced existing ablative modalities. The combination of phototherapy and chemotherapy (chemophototherapy, CPT), when carefully planned, has been shown to be an effective tumor treatment option preclinically and clinically. Chemotherapy can enhance the efficacy of PDT by targeting surviving cancer cells or by inhibiting regrowth of damaged tumor blood vessels. Alternatively, PDT‐mediated vascular permeabilization has been shown to enhance the deposition of nanoparticulate drugs into tumors for enhanced accumulation and efficacy. Integrated nanoparticles have been reported that combine photosensitizers and drugs into a single agent. More recently, light‐activated nanoparticles have been developed that release their payload in response to light irradiation to achieve improved drug bioavailability with superior efficacy. CPT can potently eradicate tumors with precise spatial control, and further clinical testing is warranted.

## 1 Introduction

Cancer is a major healthcare burden in the United States and many other countries in the world.^1^, ^2^ One in four deaths in the United States is due to cancer.^1^ Approximately 14 million new cases and eight million cancer related deaths occurred in 2012 worldwide, with lung, prostate, colorectal, stomach, and liver cancers being the most common types for men; and breast, colorectal, lung, cervix, and stomach being the most common for women.^2^

Cancer patients are most commonly treated with one or a combination of three options: chemotherapy, radiation therapy, or surgery. Surgical resection of a tumor is the preferred option, especially at early stages of disease.^3^ However, surgery is often not possible for several reasons, including the type, location, stage of cancer, and underlying diseases. For example, 95% of patients in western countries with hepatocellular carcinoma often have an underlying disease such as cirrhosis, which may make surgical resection impossible due to high risk of liver failure following surgery.^4^ In the case of pancreatic cancer, the majority of patients are unsuitable for surgical resection at the point of diagnosis due to locally advanced disease, with cancer invasion to other parts of the pancreas or nearby organs.^5^, ^6^

### 1.1 Chemotherapy

Chemotherapy—the use of chemical substance for disease treatment—is typically the main treatment for late stage cancers. It is also used in early cancer as adjunct treatment for surgery to reduce tumor size and reduce the risk of recurrence.^3^, ^7^ Traditional chemotherapeutic agents are cytotoxic and function by killing cells that are rapidly proliferating, which is a trait of cancerous cells. However, normal cells that divide relatively frequently such as cells in the bone marrow, digestive tract and hair follicles can also be harmed, resulting in side effects such as myelosuppression, nausea, vomiting and hair loss.^7^, ^8^ A few examples of chemotherapeutic agents include cytotoxic antibiotics (e.g., doxorubicin and mitomycin), alkylating agents (e.g., cisplatin and cyclophosphamide), anti‐metabolites (e.g., fluoropyrimidine, gemcitabine and methotrexate), anti‐microtubule agents (e.g., vincristine and paclitaxel), and topoisomerase I inhibitors (e.g., irinotecan and camptothecin).

Chemotherapy often requires multiple doses to be effective, which results in increasingly severe systemic toxicity and drug resistance over the treatment course. For many tumors, the efficacy of chemotherapy is often limited by the ability of the drug to accumulate in the tumor at therapeutic levels.^9^, ^10^, ^11^ Additionally, the side effects of the drugs may limit the ability for a patient to continue treatment. To address these problems, a significant amount of research has focused on developing more potent and selective anti‐cancer drugs, or developing more effective mechanisms for delivery of the anti‐cancer agents.

Nanoparticles such as liposomes, polymeric nanoparticles, drug–polymer conjugate, and micelles have been developed as drug carriers to provide selective delivery of anti‐cancer agents while minimizing toxicity to healthy organs. Nanomedicines can exploit defects in tumor microvasculature which allow for preferential tumoral accumulation based on the so‐called enhanced permeability and retention (EPR) effect.^12^, ^13^, ^14^ However, the EPR effect alone is generally an insufficient mechanism, as there are additional barriers such as high interstitial fluid pressure^15^ and heterogeneous tumor vasculature.^16^ Additionally, the rate of drug release from the carrier and uptake by cells might not be optimal. Slow drug release from the carriers has been shown to reduce the efficacy of certain nanomedicines.^17^, ^18^ For example Doxil, a FDA approved sterically stabilized liposomal formulation of doxorubicin, has been shown to have enhanced tumor drug uptake in preclinical studies. However, it does not show greater efficacy over the free drug clinically,^19^, ^20^ only a reduction in cardiotoxicity,^21^ despite its enhanced tumoral accumulation and anti‐tumor efficacy in pre‐clinical studies.^22^, ^23^ Most nanoparticles currently being used are successful by reducing side‐effects or as a means to solubilize hydrophobic drugs to induce fewer side effects.^24^ However, there has been significant preclinical work focused on developing new strategies for improving the delivery and efficacy of drugs using nanoparticles that are responsive to external stimuli such as heat,^25^, ^26^, ^27^, ^28^ light,^29^, ^30^, ^31^, ^32^, ^33^ ultrasound^34^ or tumor environment factors such as lower pH^35^, ^36^, ^37^, ^38^ or expression of certain enzymes.^39^, ^40^

### 1.2 Ablative Therapy

Ablative therapy is often used to treat tumors which cannot be easily surgically removed and which may not respond well to radiation therapy or chemotherapy.^41^, ^42^ Radiation, heat, extreme cold (cryoablation), lasers, and chemicals have been used for ablative treatments. Brachytherapy, which involves implantation of small radioactive pellets into the tumor, has been used in cervical, prostate, and breast cancers.^43^ Thermal ablation is a relatively common ablative method, and often involves radiofrequency (RF) ablation. RF ablation involves the use of a probe which can be inserted into the tumor to heat the tissue to therapeutic temperatures which can kill cancer tissues. Efficacy is often limited by uneven distribution of heat throughout the entire tumor area due to nearby blood vessels which can serve as heat sinks. Often, the margins of the tumor do not get hot enough to completely kill all the cancer cells.^42^ Researchers have developed a liposomal formulation of the anti‐cancer drug doxorubicin designed to release the drug at elevated temperatures present in an ablated tumor to enhance the efficacy of RF ablation.^44^, ^45^ The goal is to have the drug release at the margins of the tumor where the thermal ablation would not be effective on its own. A clinical trial was conducted for hepatocellular carcinoma comparing the use of RF ablation with and without these liposomes. However, the results showed there was no significant improvement with the application of the drug. The exact reason for this lack of efficacy is not clear; however, the fact that the thermosensitive liposomal formulation is unstable in serum while circulating so it may lose its cargo before reaching the tumor could be a contributing factor.^46^, ^47^, ^48^

Another thermal ablative technique is laser ablation.^49^, ^50^, ^51^ There are currently multiple laser ablation systems which have been approved, including MRI‐guided Neuroblate and Visualase for treatment of brain tumors and ultrasound or X‐ray guided Novilase for breast cancer, and they are either being developed or undergoing trials for other types of cancers. These systems use thermal feedback to guide the treatment and ensure the entire tumor can be treated. They have been shown to be effective; however, they are limited by tumor size and shape as light cannot penetrate very deep and the irregularly shaped tumors complicate the precision of the treatment.

There are also other ablative methods such as electroporation (e.g., Nanoknife),^52^, ^53^ microwave ablation,^54^ ethanol ablation,^55^ and cryoablation,^56^, ^57^ which are techniques either approved or currently being developed. Photodynamic therapy is another ablative technique that will be discussed here.

### 1.3 Photodynamic Therapy

Photodynamic therapy (PDT) is a light‐based cancer therapy and has been demonstrated to be effective as a both a curative and palliative treatment.^58^, ^59^, ^60^ Early in its development, PDT was sometimes referred to as photochemotherapy, but that term was phased out to emphasize the non‐toxic nature of the photosensitizers used.^61^ PDT involves the administration of a photosensitizer and tumor irradiation with light of a specific wavelength, sometimes in the near infrared (NIR) region of the spectrum that can more deeply penetrate tissues. Upon illumination, the photosensitizer absorbs the light which leads to a series of photochemical and photobiological reactions, including generation of reactive oxygen species such as singlet oxygen which are directly toxic to cells.^62^, ^63^ In addition to direct tumor cell killing, PDT also causes vascular damage and blood flow stasis which deprives the nutrients to tumor cells^64^, ^65^ and PDT also has also been shown to stimulate anti‐tumor immune responses.^66^, ^67^

Photosensitizers such as Photofrin are usually administrated 24 or 48 h prior to light treatment to allow for deposition of the photosensitizer in the tumor. However, there is evidence to suggest that the amount of photosensitizer in blood, rather than the amount in the tumor, at the time of irradiation determines the therapeutic outcomes.^68^, ^69^, ^70^, ^71^, ^72^ This has been demonstrated with several photosensitizers such as temoporfin,^68^, ^69^, ^71^ hypericin,^70^ and mono‐L‐aspartyl chlorin e6 (NPe6).^72^ A possible explanation is that photosensitizers near vascular endothelial cells induce tumor vasculature damage, resulting in vascular shutdown and subsequent deprivation of nutrient supply to the tumor. Pd‐bacteriopheophorbide (Tookad) is a photosensitizer that is designed for destruction of the tumor vasculature and not tumor cell kill,^73^, ^74^ and has recently been approved in Mexico for prostate cancer therapy.

Compared to conventional treatments (surgery, radiation and chemotherapy), PDT has certain advantages, including minimal normal tissue toxicity, no long‐term systemic toxicity such as immunosuppression, lack of drug resistance mechanisms, and favorable cosmetic outcomes.^58^, ^75^ PDT can be done on an outpatient basis and a single treatment is sometimes sufficient. In contrast, curative radiation therapy normally requires daily radiation for 6–7 weeks, while chemotherapy courses can last for months and surgery requires hospitalization for days to weeks.^76^

PDT has been approved for treatment of various types of cancers including head and neck tumors, basal‐cell carcinoma, cervical, endobronchial, esophageal, bladder, and gastric cancer.^77^ Originally, PDT was only used for treatment of superficial lesions in skin and luminal organs. Due to the limited light penetration through tissues, the depth of tumor destruction is generally less than 1 cm.^58^ However, since its original development there have been technical advances in interstitial and intra‐operative light delivery approaches, which allow for PDT to be used for treatment of a wider range of solid tumors,^78^ including brain,^79^ breast,^80^, ^81^ lung,^82^ pancreas,^83^, ^84^ and prostate.^85^

There are currently several photosensitizers being used for PDT clinically, including Photofrin, verteporfin, temoporfin, aminolevulinic acid (ALA) and methyl aminolevulinate (MAL). Photofrin, the partially purified form of hematoporphyrin derivative (HPD) was the first clinically approved photosensitizer for treatments of non‐small‐cell lung carcinoma, Barrett's oesophagus, endobroncheal cancer, bladder cancer, and cervical cancer.^86^ Tetrahydroxyphenylchlorin (m‐THPC, temporfin or Foscan), a member of the chlorin family, is a second generation photosensitizer approved for head and neck cancer treatment. ALA which acts as a prodrug and metabolizes to the active form protoporphyrin IX, is approved for basal‐cell carcinoma. Some new photosensitizers are undergoing clinical trials such as talaporfin (NPe6),^87^ Pd‐bacteriopheophorbide (Tookad),^88^, ^89^ and HPPH.^90^, ^91^, ^92^

While PDT has minimal long‐term systemic toxicity, it does introduce a unique side effect of sunlight toxicity. Sunlight toxicity is caused by the photosensitizer accumulating in the skin which causes the skin to be more sensitive to sunlight and other forms of ambient light. As a result, exposure to light can cause sunburn‐like symptoms and dryness. Besides the amount of light exposure, the severity of this effect is dependent on the type and dose of photosensitizer used and is also related to the clearance rate of the photosensitizer. First generation photosensitizers such as Photofrin involve high administrated doses and also have long circulation times, which increases skin accumulation, resulting in sunlight sensitivity for 4–6 weeks. Second generation photosensitizers such as temoporfin have been designed to have lower injected doses and less skin accumulation to reduce this side effect.

### 1.4 Combination Cancer Therapy

Combination therapy is frequently used clinically. For chemotherapy itself, combinations of two or more types of chemotherapeutics are often offered.^7^ As each chemotherapy has its own mechanism of action and maximal tolerated dose, combination therapy ideally leads to therapies that are more effective and have less side effects.

Chemotherapy is often offered concurrently with radiation therapy, which is termed as chemoradiotherapy (CRT). CRT has been found to be more effective than the sum of the two parts,^93^ and is often used as neoadjuvant therapy before surgical resection, with the intent of improved organ preservation and survival compared with surgical resection alone. The rationale of CRT mainly derives from two aspects.^94^ First, chemotherapy can be used as a radiosensitizer (e.g., cisplatin, 5‐fluorouracil, and taxanes) and enhance the local control, and in some cases, survival. Second, chemotherapy which acts systemically, can potentially aid in the control of distant micrometastases, which is also known as the spatial cooperation effect. Chemotherapy and/or radiation therapy are also often offered after surgical resection to prevent tumor recurrence. Clinical trial design for combination therapies can be more complex than single therapy approaches and usually involves addition of a new approach to an existing standard of care.

## 2 Combining Phototherapy with Chemotherapy

**Figure 1:**
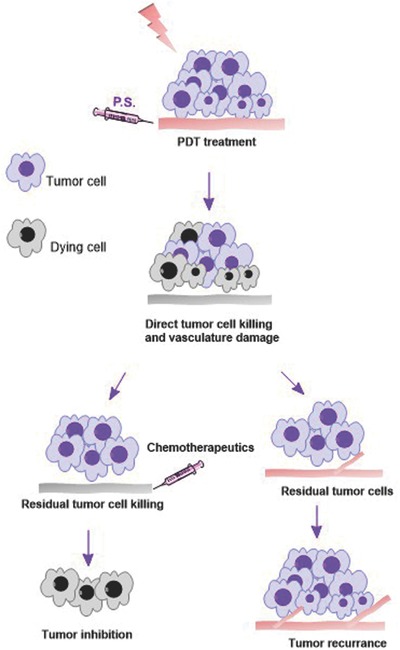
PDT combined with chemotherapy. PDT can directly kill cancer cells, damage tumor vasculature and induce anti‐cancer immunity. However, the residual cancer cells can lead to tumor recurrence due to incomplete cancer cell killing. CPT that combines PDT with subsequent chemotherapies can assist in killing the residual cancer cells and potentially lead to complete tumor inhibition.

While PDT can cure early tumors and small lesions, for advanced cancers, PDT alone cannot achieve cure and recurrence is often seen.^95^, ^96^ Many strategies have been proposed to potentiate the therapeutic outcome of PDT, including combination with anti‐cancer agents, or conjugation of photosensitizers to carrier molecules (i.e., serum proteins, peptides, antibodies) for selective cellular or vasculature‐targeted PDT.^97^, ^98^, ^99^, ^100^ The use of PDT in combination with chemotherapy (chemophototherapy, CPT) is an interesting concept which can provide a more potent treatment than using either treatment alone.^98^, ^99^ As illustrated in **Figure** **1**, PDT can directly kill tumor cells through apoptosis and necrosis and through anti‐tumor vasculature effects. However, tumor cells that survive PDT can lead to regrowth of tumor cells and tumor vessels. Introduction of chemotherapeutics concurrently can further damage tumor cells, preventing regrowth. Additionally, anti‐cancer drug themselves may generate oxidative stress, generating hydroxyl radicals which, when combined with PDT may be sufficient to induce cell cycle arrest and subsequent cytotoxic death of cancer cells.^101^, ^102^ Many preclinical studies have shown a synergistic effect of both modalities, both in vitro and in vivo (**Table** **1**) as will be discussed in the following section.

**Table 1:** Studies combining PDT and conventional chemotherapeutics

Chemo	Chemo dose (mg kg^–1^)	Chemo DLIa)	P.S.b)	P.S. dose (mg kg^–1^)	Light dose	Tumor modelc)	Ref.
Dox	3, 2 doses	24 h and 0 h	Photofrin	30	610–680 nm, 225 J cm^–2^	Lewis lung carcinoma	119
	3, 2 doses	24 h, 0 h	Photofrin	15	630 nm, 50 mW for 15 min	Walker 256 carcinosarcoma	149
	5	24 h	Photofrin	5	628 nm, 500 mW, 120 J cm^–2^	H‐MESO‐1 Mesothelioma	121
	5	48 h	Photofrin	7.5	630 nm, 200 J cm^–2^	MBT‐2 murine tumor	120
	5	16 or 40 h	ALA	N.A.	633 nm, 80 mW cm^–2^, 72 J cm^–2^	M2 mammary adenocarcinoma	122
	1 or 2	48 h before or 24 h after	AIS_2_Pc	5	670 nm, 100 mW cm^–2^, 60 J cm^–2^	L1210 leukemia, P388 lymphoma	123
	5	30 min	Photofrin	10	630 nm, <250 mW cm^–2^, 100 J cm^–2^	EMT‐6 tumors	125
	5 or 10	30 min	Photofrin	10	630 nm, <250 mW cm^–2^, 25‐200 J cm^–2^	RIF1 tumors^c‐1)^	125
MMC	3	48 h	Photofrin	7.5	630 nm, 200 J cm^–2^	MBT‐2 murine tumor	120
	1 or 5	12 h	Photofrin	20	630 nm, 135 J cm^–2^	WiDr adenocarcinoma	128
	2.5 or 5	15 min or right after	Photofrin	10	628 nm, 100 mW cm^–2^, 200–400 J cm^–2^	RIF1 tumors	131
	5	15 min	Photofrin	5 or 10	628 nm, 200 mW cm^–2^, 0–400 J cm^–2^	RIF1 tumors	133
	2.5	20 min	Hypericin^b‐1)^	1	595 nm, 100 mW cm^–2^, 120 J cm^–2^	RIF1 tumors	129
	5	15 min before or right after	m‐THPC	0.15 or 0.3	652 nm, 100 mW cm^–2^, 30–90 J cm^–2^	RIF1 tumors	130
	5	15 min	BCA^b‐3)^	20	750 nm, 0–400 J cm^–2^, 150 mW cm^–2^	RIF1 tumors^c‐2)^	130
CDDP	1 or 2	48 h before or 24 h after	AIS_2_Pc	5	670 nm, 100 mW cm^–2^, 60 J cm^–2^	L1210 leukemia, P388 lymphoma	123
	7	30 min	Photofrin	10	630 nm, <250 mW cm^–2^, 75 J cm^–2^	RIF1 tumors	125
	10	30 min	Photofrin	10	630 nm, <250 mW cm^–2^, 100 J cm^–2^	EMT‐6 tumors	125
MTX	0.2, 2 doses	24 h or 0 h before	Photofrin	30	610–680 nm, 225 J cm^–2^	Lewis lung carcinoma	119
CY	50	2 days	Verteporfin^b‐2)^	2	690 nm, 100 mW cm^–2^, 150 J cm^–2^	J774 tumor	145

^a)^Drug light interval chemotherapeutics were administered i.p. prior to irradiation;

^b)^Photosensitizers were administered i.p. 24 h prior to irradiation, except ^b‐1,b‐2)^ which were injected intravenously, and ^b‐2, b‐3)^ which were injected 15 min prior to irradiation;

^c)^Significantly enhanced anti‐tumor efficacy was achieved, except ^c‐1,c‐2)^where no significant enhancement was observed.

PDT is used as a local treatment modality whereas chemotherapy is a systematic treatment. In general, CPT would be anticipated to function as more potent form of PDT. For instance, drug diffusion through solid tumors beyond the confines of the irradiated volume might enable a larger ablation zone. However, the fact that chemotherapy acts systemically can potentially aid in the eradication of distant micrometastases, similar to the spatial cooperation effect in CRT. Moreover, preclinical and clinical studies have shown that PDT can induce anti‐tumor immunity and may be beneficial for the control of distant tumors.^66^, ^103^ PDT treatment of multifocal angiosarcoma of the head and neck led to tumor regression of distant untreated tumors.^104^ PDT combined with chemotherapy can potentially lead to a more effective systemic anti‐cancer treatment option. Such combination treatments have been tested clinically and demonstrated an enhanced anti‐tumor response compared to either PDT or chemotherapy alone.^95^, ^105^, ^106^, ^107^

Doxorubicin (Dox), mitomycin (MMC), and cisplatin (CDDP) have been most frequently studied in combination with PDT in preclinical CPT studies. Other chemotherapy drugs used for CPT include methotrexate and gemcitabine.

### 2.1 Doxorubicin (Dox)

Doxorubicin (Dox, or adriamycin) is an anthracycline antibiotic used clinically for a wide range of solid tumors and hematological malignancies, including advanced breast cancer, small cell lung cancer, AIDS‐related Kaposi's sarcoma, acute leukemia, and lymphomas and myeloma.^108^ Dox functions in several ways including inhibiting DNA synthesis via intercalation, inhibition of topoisomerase II,^108^, ^109^, ^110^, ^111^free radical formation,^112^, ^113^, ^114^, ^115^ and lipid peroxidation.^116^, ^117^ Dox is considered to be one of the most potent anti‐cancers drugs, with cardiotoxicity being the main side effect.^108^, ^118^

Dox has been shown to potentiate PDT responses in multiple studies.^119^, ^120^, ^121^, ^122^, ^123^, ^124^ Cowled et al. first studied the interaction between PDT (with the HPD photosensitizer) and Dox both in vitro and in vivo.^119^ Two doses of Dox given at the time of HPD injection and at the time of irradiation resulted in significantly increased survival while injection of Dox 24 and 48 h after irradiation resulted in slight potentiation in mice bearing Lewis lung carcinoma. Compared to mice receiving HPD alone, those which received Dox concurrently with HPD exhibited more intense HPD fluorescence, suggesting that enhancement of tumor uptake of photosensitizers could be one mechanism of how Dox potentiates the PDT response. Interestingly, in vitro studies showed that Dox inhibited HPD uptake in a concentration dependent manner, in both Raji and Lewis lung carcinoma cells. Thus, the mechanism of the enhanced efficacy is not fully understood. The enhancement of efficacy may be associated with the sum of the damages induced by both modalities.^120^ The cyctotoxic effect of Dox on capillaries could damage the tumor cells and tumor microvasculature, which is further damaged by PDT.

A similar study demonstrated that Dox enhanced the PDT response in mesothelioma cells both in vitro and in vivo.^121^ Co‐injection (intraperitoneal injection, i.p.) of Dox and Photofrin 24 h prior to irradiation resulted in 100% necrosis of mesothelioma tumors 5 days post‐treatment and no tumor regrowth after 30 days. PDT alone, however, resulted in 50% decrease of the tumor surface area, while regrowth was seen on day 30. Dox alone with or without light illumination had no effect in delaying tumor growth. In vitro studies on H‐MESO‐1 cells confirmed the addition of DOX can enhance the effectiveness of PDT.

One benefit of using such combination therapy is the ability to reduce the effective dose of Dox. Very low doses of Dox (1 or 2 mg kg^–1^) combined with a second generation photosensitizer, aluminum disulfonated phthalocyanine (AIS_2_Pc) could be used to treat mice inoculated with L1210 leukemia and P388 lymphoma.^123^ The combination of 1 or 2 mg kg^–1^ Dox and PDT significantly increased the median survival time compared to PDT (100 mW cm^–2^, 60 J cm^–2^) or Dox alone in both tumor models. A possible explanation for this enhancement was the sum of damages induced by both Dox cytotoxic effect and PDT effect. In contrast to the results from Cowled et al.,^119^ the sequence of Dox injection (before or after illumination) was not found to have a significant effect on efficacy, as Dox given 1 day after PDT treatment was equally effective.

In an EMT‐6 murine tumor model, Dox i.p. injected 15 min before illumination with HPD given 24 h earlier resulted in significant enhancement of efficacy.^125^ However, these effects were not observed in all tumor models. When tested on a radiation‐induced fibrosarcoma (RIF‐1) tumor model, no similar delay in tumor growth was seen as RIF‐1 tumors were found to be insensitive to both Dox and PDT.

### 2.2 Mitomycin (MMC)

Mitomycin (MMC) is an antibiotic with a potent anti‐tumor effect through inhibition of DNA synthesis.^126^ Ma et al. investigated the effect of MMC on photodynamic therapy in cultured WiDr human colon adenocarcinoma cells.^127^ An almost nontoxic dose of MMC (0.01 μg/ml) reduced the D_50_ (50% of cell survival) by a factor of 2.5 while a nontoxic PDT treatment could also enhance the cytotoxic effect of MMC. The sequences of the two treatments was found to be important. MMC and Photofrin given simultaneously followed by light irradiation was more effective than MMC given after irradiation. In a follow‐up study with WiDr xenograft, it was reported that the anti‐tumor activity of PDT in combination with MMC had a greater effect on slowing tumor growth than either treatment alone.^128^ This enhancement was more prominent when PDT was combined with a low dose of MMC (1 mg kg^–1^). It was suggested that the increased susceptibility to PDT may be due to the MMC‐induced accumulations of cells in S‐phase.^127^, ^128^

Combining MMC with PDT enables the reduction of both light dose and photosensitizer dose.^129^, ^130^ Geel et al. studied the combination of MMC and PDT with three different photosensitizers, meso‐tetrahydroxyphenylchlorin (m‐THPC), bateriochlorin α (BCA) and Photofrin.^130^ It was demonstrated that m‐THPC in combination with MMC resulted in a significant increase in tumor response and the light dose required for 50% cures was reduced by half (from 124 J cm^–2^ to 67 J cm^–2^). m‐THPC‐mediated PDT alone resulted in longer regrowth times and more cures than PDT with Photofrin. In vivo and in vitro cell survival studies compared the contribution of direct tumor cell death and indirect vascular effects by determining the cell survival at different times after PDT treatment. There was little direct cell killing (immediately after treatment) for both Photofrin and m‐THPC, and the tumor cell survival decreased as the interval between treatment and excision increased. Compared with Photofrin in combination with MMC, m‐THPC PDT allows for lower light and m‐THPC doses. Notably, MMC given immediately after irradiation was as effective as given 15 min prior to m‐THPC PDT, while combination of BCA PDT with MMC did not result in a significant increase in survival when given 15 min prior to irradiation.

Bass et al. studied the enhancement of interstitial PDT by MMC in a RIF‐1 mouse tumor model.^131^ Interstitial PDT with MMC improved the tumor response compared with MMC or light treatment alone. The light dose required for 50% cure could be reduced by a factor of 2 when MMC was given before PDT irradiation. Since MMC is also a bioreductive drug which has increased cytotoxicity in hypoxia cells,^132^ the mechanism for this enhancement was suggested to be the hypoxia induced by the PDT which enhanced the cytotoxicity of MMC. Similar to the finding from Ma et al., MMC given immediately after illumination did not increase the effects of interstitial PDT. The same group later documented a preclinical and clinical study of enhancement of photodynamic therapy using MMC.^133^ They demonstrated that similar results could be obtained using lower photosensitizer and light doses when MMC is included.

### 2.3 Cisplatin (CDDP)

Cisplatin (cis‐diammine dichloroplatinum, CDDP) is widely used in endocrine‐related cancers such as ovarian and testicular carcinomas.^134^ It works by interacting with DNA to form DNA adducts which inhibit DNA replication.^135^ However, cisplatin is also associated with adverse traits such as nephrotoxicity and drug resistance.^136^

PDT has been shown to sensitize ovarian cancer cells to chemotherapy and biological agents.^137^, ^138^ For this reason, combining PDT and cisplatin could be used to enhance the effects of cisplatin while reducing the effective dose.

Crescenzi et al. investigated the cytotoxicity of low‐dose cisplatin with indocyanine green (ICG)‐mediated PDT on MCF‐7 cells which demonstrated a mutual reinforcement of both modalities.^139^ PDT altered the expression of proteins related to cell death, with a reduced expression of Bcl‐2 and increased expression of Bax. Flow cytometry revealed that PDT killed mostly G_1_‐phase cells, whereas cisplatin targeted mostly S‐phase cells. A follow up study examined the response of non‐small‐cell lung cancer H1299 cells to the combination of PDT and CDDP.^140^ Various treatment combinations could result in therapeutic effects ranging from additive to synergistic. Similar to their previous study, PDT with Photofrin targeted G_0_–G_1_ cells and led to an accumulation of S phase cells. In contrast, low dose CDDP targeted S phase cells, resulting in an accumulation of cells in G_0_–G_1_ phase. This disjointed phase‐related cytotoxic activity of PDT and CDDP may account for the synergistic outcome of the combinatorial therapy.

The combination of low dose CDDP with aluminum disulfonated phthalocyanine (AIS_2_P_C_) mediated PDT was studied in mice inoculated with L1210 leukemia and P388 lymphoma.^123^ CDDP given 48 h prior to PDT illumination significantly prolonged the survival compared with either PDT or CDDP alone. CDDP given 24 h after illumination was equally effective to CDDP given 48 h prior to illumination. The combination therapy would enable the use of reduced effective doses of CDDP which lowers the toxic side effects.

### 2.4 Other Chemotherapy Drugs

Other chemotherapy drugs have been used in combination with PDT. Cyclophosphamide (CY) is an alkylating agent for cancer treatment which normally causes immunosuppression.^141^ Interestingly, CY at low doses can act as an immunostimulatory agent.^142^, ^143^, ^144^ Castano et al. reported a study combining BPD‐mediated PDT and low‐dose CY on a J774 reticulum cell sarcoma xenograft.^145^ BPD‐PDT in combination with low dose CY (50 mg kg^–1^) resulted in a 70% tumor inhibition. However, high‐dose CY (150 mg kg^–1^) in combination with PDT was not effective, resulting in no significance over mice receiving no treatment. PDT and CY alone (both high and low dose) led to no permanent cure. Cured mice were inoculated a second time with J774 cells but tumor growth failed in 71% of mice. Tumor specific T‐cells were identified in the cured mice. The fact that only low dose CY combination resulted in long term cure suggested that CY was not acting as traditional cytotoxic agent, but as an immunostimulatory agent.

Other combinations with methotrexate (MTX) and gemcitabine have also been investigated. Cowled et al. demonstrated that MTX potentiated HPD‐mediated PDT on mice bearing Lewis lung carcinoma xenografts.^119^ Anand et al. studied the combination of low‐dose MTX with ALA‐based PDT on skin carcinoma cells in vitro and in vivo.^146^ It was demonstrated that low dose MTX pretreatment of monolayer cultures enhanced the intracellular photosensitizer levels by a factor of 2 to 4. MTX pretreatment synergistically enhanced ALA‐mediated photodynamic killing. In vivo studies further demonstrated that MTX preconditioning enhanced protoporphyrin IX (PpIX) accumulation in three models.

Gemcitabine is a nucleoside analog used as a first‐line treatment of locally advanced or metastatic adenocarcinoma of the pancreas.^147^ Sun et al. studied the synergistic effects of HPPH‐mediated PDT and gemcitabine in pancreatic cancer cell lines (PANC‐1, MIA Paca‐2, BxPC‐3).^148^ It was demonstrated that HPPH‐PDT can induce cell death in a dose dependent manner. Combining gemcitabine with HPPH‐PDT led to a synergistic cytotoxic effect. Notably, gemcitabine given before or after PDT had no difference on the cytotoxic effect of the combination therapy. Crescenzi et al. studied the combination effect of gemcitabine and Photofrin‐mediated PDT effect in metastatic non‐small cell lung cancer cells (H1299).^140^ Depending on the different treatment combinations, the therapeutic outcomes ranges from additive to synergistic. However, the therapeutic reinforcement was not as pronounced as CDDP plus PDT. Unlike CDDP which targets cells in S phase and caused disjointed phase‐related cytotoxic activity, gemcitabine targeted G_0_–G_1_ cells (same as PDT) and exhibited no such disjointed phase‐related cytotoxic activity.

### 2.5 Clinical Combinatorial Studies

Several clinical studies on combining chemotherapies and photodynamic therapy have been reported. Jin et al. reported the combined treatment of HPD‐mediated PDT and chemotherapy for patients with advanced cardiac cancers.^105^ Chemotherapy drugs used were Tegafur/Uracil (TFU) and MMC. It was demonstrated that PDT with chemotherapy resulted in a significantly higher rate of complete remission (19.5% versus 5.5%) than chemotherapy alone. More patients achieved remission after PDT‐combined chemotherapy with a prolongation of survival. This demonstrated that CPT was a safe treatment for patients with advanced cardiac cancer.

Another study from Li et al. compared the short‐term curative effect of PDT, PDT combined with chemotherapy (5‐FU and CDDP) and chemotherapy alone on patients with advanced esophageal cancer.^95^ There was no statistically significant difference in effectiveness of PDT alone, the complex treatment and chemotherapy (85.2%, 90.9%, and 63.3%). However, the 2 year survival rate (29.6%, 54.5% and 16.7%) and medium survival time (stage III 13 months, 22 months, 10 months; stage IV 7 months, 5 months, 4 months) of people receiving combination therapy were significantly greater. It was concluded that PDT combined with chemotherapy for advanced esophageal cancer was superior to PDT and chemotherapy alone.

PDT plus oral fluoropyrimidine, S‐1 has been tested clinically for patients with unresectable hilar cholangiocarcinoma (UHC), a condition for which PDT has been used as a palliative treatment.^106^ Patients were treated with either Photofrin‐mediated PDT plus S‐1 or PDT alone. Compared to PDT alone, PDT combined with S‐1 was found to be well tolerated and associated with significant improvement of overall survival (1 year survival rate, 76.2% versus 32%; median survival, 17 months versus 8 months) and prolonged progression‐free survival (median survival 10 months versus 2 months).

Another clinical study on PDT plus systemic chemotherapy for treatment of advanced hilar cholangiocarcinoma was conducted.^107^ In this study, patients were treated with PDT plus chemotherapy (gemcitabine or gemcitabine with cisplatin) or PDT alone. PDT plus chemotherapy resulted in significantly longer survival compared to PDT alone (538 days versus 334 days).

## 3 Combining Phototherapy with Novel Anti‐Cancer Agents

**Figure 2:**
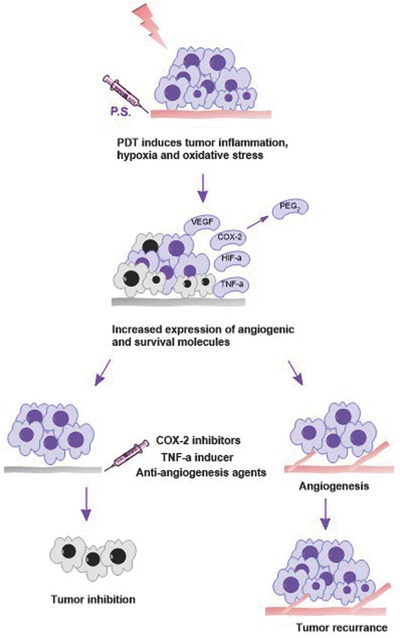
Combining inhibitors to angiogenenic and survival molecules with PDT. PDT increases the expression of many transcription factors and cytokines which are related to oxidative stress and inflammation. Angiogenenic and survival molecules are subsequently increased following PDT treatment. These molecules may induce tumor recurrence. The combination of PDT with inhibitors to these up‐regulated molecules can potentiate responses.

PDT induces apoptosis and necrosis in treated tumors, causes damages to the microvasculature and leads to inflammation, hypoxia and oxidative stress. These processes are correlated with the up‐regulation of angiogenesis factors such as hypoxia‐inducible factor‐1 (HIF‐α), vascular endothelial growth factor (VEGF), tumor necrosis factor (TNF‐α), interleukin‐1β (IL‐1 β), prostaglandin E_2_ (PEG_2_), cyclooxygenase‐2 (COX‐2), survivin and matrix metalloproteinases in PDT‐treated tumors.^150^, ^151^, ^152^ These molecules may be related to tumor recurrence. The combination of PDT with inhibitors to these angiogenesis factors and survival molecules can improve the anti‐tumor responses. As illustrated in **Figure** **2**, PDT treatment increases the expression of angiogenic and survival molecules (VEGF, COX‐2, HIF‐α and survivin) which could lead to angiogenesis and tumor recurrence. The combination of COX‐2 inhibitors, TNF‐α inducer, or anti‐angiogensis agents can potentiate the PDT response and lead to more efficient tumor inhibition. **Table** **2** summarizes the studies combining these novel anti‐cancer agents with PDT, which significantly potentiate the anti‐tumor responses.

**Table 2:** Studies combining PDT and novel anti‐cancer agents

Anti‐cancer agentsa)	Mechanism	P.S.b)	P.S. dose	Light wavelength and dose	Tumor modelc)	Ref.
NS‐398	COX‐2 inhibitor	Photofrin	5 mg kg^–1^	630 nm, 75 mW cm^–2^, 200 and 300 J cm^–2^	RIF tumor	159
Celecoxib or NS‐398	COX‐2 inhibitor	Photofrin	5 mg kg^–1^	630 nm, 75 mW cm^–2^, 200 J cm^–2^	BA carcinoma	150
2,5‐dimethyl celecoxib	Celecoxib analog	Photofrin	5 mg kg^–1^	630 nm, 75 mW cm^–2^, 200 J cm^–2^	BA carcinoma	152
Nimesulide	COX‐2 inhibitor	Photofrin	10 mg kg^–1^	630 nm, 80 mW cm^–2^, 150 J cm^–2^	C‐26 colon adenocarcinoma	187
NS‐398	COX‐2 inhibitor	NPe6	5 mg kg^–1^, 1 or 2 doses	664 nm, 9 mW cm^–2^, 61 J cm^–2^	Colon‐38 cells	160
DMXAA	induce TNF‐a synthesis	Photofrin	1.5 mg kg^–1^	630 nm, 75 mW cm^–2^, 135 J cm^–2^	RIF‐1 tumors	172
DMXAA	TNF‐a synthesis	HPPH	0.4 μmol kg^–1^	665 nmm, 112 mW cm^–2^, 48 J cm^–2^	C‐26	173
DMXAA	TNF‐a synthesis	HPPH	0.4 μmol kg^–1^	665 nm, 112 mW cm^–2^, 48 J cm^–2^	C‐26 colon carcinoma	174
rHuTNF‐α	Induces hemorrhagic necrosis	Photofrin	2.5 mg kg^–1^	630 nm, 160 mW cm^–2^, 288 J cm^–2^	SMT‐F tumors	171
IM862 or EMAP‐II	VEGF inhibitor	Photofrin	5 mg kg^–1^	630 nm, 75 mW cm^–2^, 200 J cm^–2^	BA carcinoma	151
TNP‐470	synthetic anti‐angiogenesis agent	Temoporfin	0.25 mg kg^–1^	690 nm, 100 J cm^–2^	LNCaP tumor	179
PD166285 and PD173074	Synthetic RTK inhibitors	HPPH	0.3 mg kg^–1^	665 nm, 135 J cm^–2^, 75 mW cm^–2^	Murine 16c tumor	183
SU5416 and SU6668	Synthetic RTK inhibitors	Hypericin	2 mg kg^–1^	60 mW cm^–2^, 47.7 J cm^–2^	CNE2 tumor	180
Avastin	VEGF monoclonal antibody	Hypericin	5 mg kg^–1^	125 mW cm^–2^, 150 J cm^–2^	MGH bladder cancer	184
Cetuximab	Monoclonal antibody	Hypericin	5 mg kg^–1^	690 nm, 100 mW cm^–2^, 120 J cm^–2^	MGH bladder cancer	185
VEGFR‐1 and VEGFR‐2	Antibodies against VEGFR	Photofrin	2 mg kg^–1^	635 nm, 80 J cm^–2^	U87 glioblastoma	186

^a)^Anti‐ cancer agents above required multiple doses to achieve significant enhancement of anti‐tumor efficacy, except for DMXAA and rHuTNF‐α which were only administered once;

^b)^P.S: Photosensitizers. Photofrin was normally administered 24 h prior to illumination, while temoporfin was intravenously administered 1 h prior to illumination; hypericin was given 2–6 h prior to illumination;

^c)^Combination of PDT and novel anti‐cancer agents led to significant enhancement of anti‐tumor efficacy in all the tumor models tested.

### 3.1 COX‐2 Inhibitors

Cyclooxygenase (COX) is an enzyme responsible for the metabolic conversion of arachidonic acid to prostaglandins.^153^ COX‐2 inhibitors are a type of non‐steroidal anti‐inflammatory drugs (NSAID). Increasing evidence shows that COX‐2 inhibitors may be novel candidates for chemotherapy^154^ and have been evaluated for treatment of various cancers.^155^, ^156^, ^157^ COX‐2 inhibitors such as celecoxib can inhibit tumor angiogenesis and suppress tumor growth.^158^ Ferrario et al. reported that PDT induced prolonged expression of COX‐2, but not COX‐1 in mouse sarcoma and carcinoma cell lines. PEG_2_ expression was also increased in PDT treated cells.^159^ The COX‐2 inhibitor, NS‐398, significantly enhanced tumor responsiveness to PDT in vitro and in vivo.^159^, ^160^ Multiple doses of NS‐398 combined with Photofrin‐mediated PDT enhanced the long‐term control of mice bearing RIF tumors, with over 50% cure at 200 J cm^–2^ and over 70% cure at 300 J cm^–2^, while PDT alone had no cure at 200 J cm^–2^ and 20% cure at 300 J cm^–2^. Systemic administration of NS‐398 reduced both PEG_2_ and VEGF in PDT treated RIF tumors.

The same group later on examined how the combination of PDT and COX‐2 inhibitors, celecoxib and NS‐398, improved the treatment responses in mouse BA mammary carcinoma.^150^ Mice bearing BA tumors treated with PDT and celecoxib or NS‐398 had significant improvement in long‐term tumor free survival compared with those treated with PDT or COX‐2 inhibitors alone. Administration of COX‐2 inhibitors was found to inhibit the PDT‐induced expression of prostaglandin E_2_ (PEG_2_) and VEGF in treated tumors. COX‐2 inhibitors increased the cytotoxicity and apoptosis in PDT‐treated BA mammary cells in vitro, but not in PDT‐treated tumors in vivo. The combination also decreased the in vivo expression of pro‐inflammatory factors such as TNF‐α and IL‐1β in treated tumors. It was thus concluded that PDT responsiveness can be improved by administration of COX‐2 inhibitors via inhibition of the expression of antigenic and inflammatory molecules induced by PDT.

Combining COX‐2 inhibitors with PDT improved the tumoricidal activity of PDT. However, it is noted that cardiovascular side effects such as heart attack and stroke are associated from long‐term use of COX‐2 inhibitors.^161^, ^162^ It is not clear whether extended use of COX‐2 inhibitor would be necessary in order to achieve significant increase of anti‐tumor efficacy. Thus alternative inhibitors that mimic the anti‐tumor effect of COX‐2, but do not share these cardiovascular risks are desirable. 2,5‐dimethyl celecoxib (DMC), is a non‐cyclooxygenase‐2 inhibitor analog of celecoxib that can mimic the anti‐tumor effect of celexib.^163^ Ferrario et al. discovered that DMC enhanced PDT tumoricidal responsiveness without altering COX‐2 activity.^152^ DMC and celecoxib reduced pro‐survival protein survivin expression induced by PDT, enhanced the endoplasmic reticulum stress response of PDT and increased apoptosis and cytotoxicity in BA cells. DMC was demonstrated to improve PDT by increasing apoptosis and cyctotoxicity without modulating COX‐2 catalytic activity. Since long‐term use of celecoxib may increase the cardiovascular side effects of COX‐2 inhibitors, DMC which lacks COX‐2 inhibitory activity appears to be a promising replacement of celecoxib.

Similar studies from Makowski et al. reported the enhanced anti‐tumor effects of the combinatorial treatment of PDT and COX‐2 inhibitors as well. Photofrin–PDT induced the expression of 5 of 140 stress related genes and 1 of the 5 encodes for COX‐2.^164^ In contrast to the finding from Ferrario et al. which demonstrated that combination of COX‐2 inhibitors potentiated PDT responses in vitro,^150^ addition of COX‐2 inhibitors, NS‐398, rofecoxib or nimesulide before or after PDT did not potentiate PDT responses in C‐26 cells. The COX‐2 inhibitor nimesulid given after, but not before PDT irradiation, significantly enhanced the anti‐tumor efficacy on C‐26 tumor bearing mice, resulting in complete response in the majority of (60% cure) treated mice.

### 3.2 DMXAA (ASA404)

5,6‐dimethylxanthenone (DMXAA or ASA404), flavone acetic acid analog, is a potent vascular disrupting agent that has completed multiple clinical trials, including a large scale phase III trial of carboplatin and paclitaxel with or without ASA404 in advanced non‐small‐cell lung cancer (NSCLC).^165^, ^166^, ^167^, ^168^, ^169^

Preclinical studies have demonstrated that DMXAA efficacy in tumor models is the result of stimulation of TNF‐α generation which leads to tumor hemorrhagic necrosis.^170^ TNF‐α was shown to be able to potentiate the antitumor responses of PDT.^171^ Bellnier et al. first demonstrated that DMXAA can selectively enhance Photofrin‐based PDT in RIF‐1 tumor bearing mice.^172^ The enhanced PDT effect appeared to be dependent on TNF‐α induction, because neutralizing antibodies to TNF‐α reduced the antitumor efficacy to control levels. Notably, the enhanced anti‐tumor efficacy of this combinatorial treatment was strongly dependent on the sequence and time of DMXAA administration. DMXAA given prior to illumination significantly enhanced PDT response while given after PDT resulted in no enhancement. DMXAA administration 1–3 h before illumination was more effective, and 2 h before illumination was particularly effective. Histological examination revealed significantly reduced blood vessel counts and increased necrosis in tumors treated with combinatorial therapy. The antitumor efficacy of the combination treatment appeared to be greater than a simple additive effect.

Seshadri et al. further studied the tumor vascular response to HPPH‐mediated PDT in combination with DMXAA in mice bearing C‐26 tumors.^173^ The combination therapy provided therapeutic synergy and selective antitumor activity, resulting in over 70% cure (90 days study period) whereas PDT alone resulted in no cure and DMXAA led to 5% cure. Enhanced permeability (4 h post treatment) due to endothelial cell damage followed by reduced blood flow (24 h post treatment) was observed in high‐dose DMXAA or the combination of PDT with low dose DMXAA. It was suggested that the combination of DMXAA and PDT resulted in a more tumor‐selective vascular response in the combinatorial treatment.

The combination of DMXAA with PDT also demonstrated significantly increased selectivity and reduced side effect compared to PDT alone in mice bearing C‐26 tumors.^174^ It is known that PDT is more effective at low fluence rates;^175^, ^176^ however, such regimes require longer treatment times (152 min, 128 J cm^–2^ at 14 mW cm^–2^ using HPPH) which is not ideal in clinical settings. When combined with DMXAA, only 7 min (lower light dose, 48 J cm^–2^ at 112 mW cm^–2^) light treatment was needed to reach similar anti‐tumor efficacy of 60% cures. The DMXAA + short‐duration PDT treatment markedly reduced the peritumoral edema and had lower rates of phototoxicity compared to long‐duration PDT monotherapy. The increased induction of TNF‐α and IL‐6 and extensive vascular damage of the combinatorial therapy could be associated with the enhanced efficacy.

### 3.3 Anti‐Angiogenesis Agents

PDT‐induced vascular damage and hypoxia will subsequently stimulate the expression of VEGF,^177^, ^178^, ^179^, ^180^ an endothelial cell‐specific mitogen that stimulates angiogenesis and promotes metastasis in solid tumors.^181^, ^182^ Ferrario et al. demonstrated that antiangiogenic treatment enhances PDT responsiveness in mouse BA mammary carcinoma.^151^ PDT plus multiple doses of the anti‐angiogenesis agents EMAP‐II or IM862 resulted in a cure rate 89% and 78%, respectively, whereas PDT alone with a light dose of 200 J cm^–2^ at a fluence rate of 75 mW cm^–2^ led to a cure rate of 39%. Similar studies demonstrating the benefits of combining antiangiogenic agents with PDT utilizing synthetic tyrosine kinases (RTKs) inhibitors such as PD166285 and PD173074,^183^ SU5416, and SU6668^180^ have also been reported.

In addition to controlling local tumor growth, PDT plus the anti‐angiogenesis agent TNP‐470 was also effective in controlling metastases in an orthotopic prostate cancer mouse model.^179^ Notably, the enhanced efficacy of TNP‐470 was treatment sequence dependent as TNP‐470 given before PDT was less effective at local tumor control than given after PDT irradiation.

Other studies involving VEGF‐specific monoclonal antibodies such as Avastin (bevacizumab),^184^ Erbitux,^185^ and MF1 and DC101^186^ in combination with PDT have also demonstrated enhancement in antitumor response.

## 4 Nanoparticle‐Mediated CPT

### 4.1 Chemo‐Photodynamic Systems

The enhanced permeabilization and retention (EPR) effect is the result of abnormal tumor vasculature which allows for the enhanced accumulation of macromolecules and nanoparticles. The EPR effect is considered an important factor in the design of nanoparticles such as liposomes, polymers, micelles, and protein drug conjugates for cancer therapy. However, some concerns exist about the heterogeneity of the EPR effect on large or late‐stage tumors, or in clinical situations.^12^, ^13^, ^188^ In these cases, tumors exhibit lower vascular density, especially in the central or hypoxic parts of the tumor. Thus, the EPR effect alone is ineffective in allowing for the delivery of therapeutic agents throughout the tumor.

PDT has been demonstrated to permeabilize the tumor vasculature and enhance the delivery of nanoparticles.^189^, ^190^, ^191^, ^192^, ^193^ The mechanism of PDT‐induced vascular permeabilization is believed to be the direct attack of cytotoxic singlet oxygen generated by PDT, which leads to damage of the vascular endothelial cells and formation of endothelial intercellular gaps.^190^, ^193^ Other mechanisms such as leukocyte recruitment also play important roles for the PDT‐induced permeabilization, as inhibition of leukocyte interaction with endothelial cells led to significantly reduced extravasation of macromolecules.^194^ PDT can activate leukocytes^195^ and leukocytes are known to induce microvessel permeability by many ways, including secretion of chemokines and adhesion dependent mechanisms.^194^, ^196^

**Figure 3:**
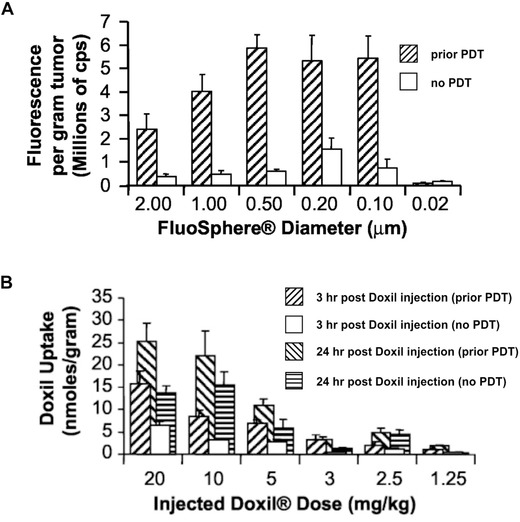
PDT as a means to enhance tumoral uptake of nanoparticles. A) Tumor uptake of fluorescent microspheres as a function of particle size in a contralateral C‐26 tumor model with only one of the tumors treated with PDT. B) Doxorubicin concentration in tumors after intravenous injection of 2.5–20 mg kg^–1^ Doxil. Tumors were treated 24 hours prior to drug administration with PDT using 0.4 μmol kg^–1^ HPPH. Reproduced with permission^189^ Copyright 2003, American Association for Cancer Research.

Snyder et al. demonstrated that PDT using HPPH can be used as a means to facilitate the delivery of macromolecular agents.^189^ With a 24 h drug–light interval, PDT regimens that used low fluence rates were demonstrated to be more successful, with the highest accumulation of fluorescent microspheres occurred within the range of 48–88 J cm^–2^ and 14–28 mW cm^–2^. PDT‐enhanced EPR effects have been shown to be more effective for nanoparticles and large molecules such as dextrans compared to small molecules^189^, ^190^, ^197^ and in this study PDT enhanced accumulation of 0.1 to 2 μm particles while small 0.02 μm particles did not show enhanced accumulation after PDT (**Figure** **3**A). PDT improved the tumor uptake of Doxil 2–3‐fold (Figure 3B). This increased tumor uptake was dependent on the time interval between completion of PDT and administration of Doxil, and Doxil administrated immediately after PDT resulted in the most accumulation. The therapeutic efficacy of the combination of HPPH‐PDT and Doxil significantly enhanced tumor control compared to Doxil or HPPH‐PDT alone.

Using Visudyne‐mediated PDT at short drug light interval (15 min), Cheng et al. demonstrated that low‐dose PDT (35 mW cm^–2^, 10 J cm^–2^) selectively enhanced the tumoral uptake of pegylated liposomal doxorubicin ≈2 fold, whereas surrounding normal lung tissue did not show improved drug uptake in a rat model of lung sarcoma tumors.^192^ Follow‐up studies on different tumor types grown on rodent lungs revealed that PDT lead to a significant increased ratio of tumor to lung tissue drug uptake for all three tumor types (sarcoma, mesothelioma, and adenocarcinoma).^198^ However, tumoral drug uptake varied between different tumor types and paralleled tumor vascular density.

**Figure 4:**
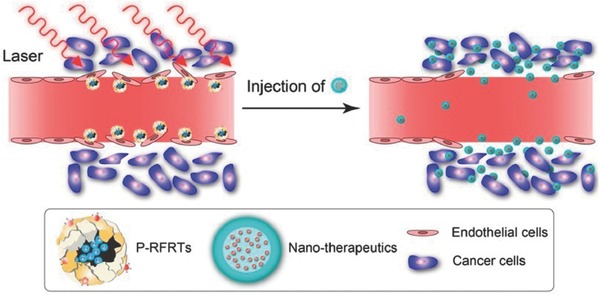
Molecularly targeted PDT for enhanced delivery of nanoparticles to tumors. P‐RFRTs are first administered and located to tumor endothelium through RGD‐integrin interactions. Following irradiation, P‐RFRTs‐mediated PDT led to enlarged or newly formed endothelial gaps and increased vascular permeability, facilitating the massive increase of accumulating nanoparticles. Reproduced with permission.^199^ Copyright 2003, American Chemical Society.

Recently, the photosensitizer ZnF_16_Pc was loaded into RGD‐modified ferritin (RFRT) and used as a smart carrier that specifically delivery PDT‐induced singlet oxygen to tumor endothelium (**Figure** **4**).^199^ RFRTs have a strong binding affinity toward integrin αvβ3 that are overexpressed on neoplastic endothelial cells. Following irradiation (24 h drug‐light interval), the tumoral accumulation of albumins was significantly enhanced and interestingly, there was a change in their distribution pattern: albumins penetrated much deeper into the irradiated tumors in the while in the non‐irradiated tumors, the albumins were found only in the tumors periphery. The enhanced EPR effect by PDT was found to dependent on the fluence rate used, with 14 mW cm^–2^ (fluence 25 J cm^–2^) demonstrated to be optimal. This concept was verified in 4T1, U87MG, MDA‐MB‐4355, and PC‐3 tumor xenograft models using various nanoparticles including albumins, quantum dots, and iron oxide nanoparticles. This treatment was reported to enhance tumoral accumulation of quantum dots by as much as 20.8‐fold. When combined with Doxil followed by irradiation, significant improved tumor growth inhibition was shown in animals received P‐RFRTs + Doxil + irradiation compared to the corresponding controls.

Related work utilizing Panitumumab (Pan)‐IR800 conjugate has been reported by Sano et al.^200^ Panitumumab, a FDA‐approved monoclonal antibody directed at HER1 was conjugated to IR700 for photoimmunotherapy. Following irradiation, the antibody‐photosensitizer conjugate led to a profound increase of vascular permeabilization. Up to 24‐fold greater tumor uptake of nanoparticles in the irradiated tumors compared to the controls was observed. This concept was termed “super‐enhanced permeability and retention” (SUPR) and was also demonstrated with a liposome containing daunorubicin (DaunoXome), in which antibody‐photosensitizer with NIR irradiation allowed for 12.3‐fold higher and more widely distributed drug signal, and significant enhanced tumor growth inhibition in A431 tumors.

Most PDT‐induced vascular permeabilization studies have focused on low fluences and fluence rates to avoid the vessel‐occlusive effect of PDT. However, with a higher fluence rate (200 mW cm^–2^, 150 J cm^–2^), it has recently been demonstrated that a 5‐fold and 3‐fold increase of tumoral Dox accumulation 4 h and 24 h post irradiation is also possible.^201^ This suggests at least some tumor vessels can still function after PDT treatment at high fluence and fuence rates. **Table** **3** summaries some nanoparticles used for CPT that have demonstrated enhancement of anti‐tumor efficacy compared to chemotherapy or photodynamic therapy alone.

**Table 3:** Tumor inhibition studies with nanoparticle‐mediated CPT

Strategya)	Enhanced tumor drug uptake	Quantitative tumor inhibition outcomeb)	Ref
HPMA copolymer‐Dox conjugates(P‐A) combined wth HPMA copolymer‐**meso‐chlorin e6** conjugate(P‐C)	–	P‐A + P‐C with light significantly enhanced tumor inhibition (cures achieved) compared to P‐A or P‐C with light (0% cure)	202, 203
SN‐38‐loaded **chlorin**‐core star block copolymer(SN‐38/CSBC micelles)	–	SN‐38/CSBC synergistically inhibit tumor growth (60% cure,3 doses) vs monotherapies (0% cure)	204
Dox‐**methylene blue** loaded nanoparticles(D‐M‐NP)	–	D‐M‐NP enhanced tumor growth inhibition vs monotherapies	205
Cisplatin and **pyro‐lipid** core–shell nanoparticles(NCP@pyrolipid)	–	Combined therapy led to 83% reduction of tumor volume; mono‐chemotherapy or mono‐PDT did not inhibit tumor growth	206
Liposomal paclitaxel (PL‐PTX) combined with liposomal **photoporphyrin IX dimethyl ester**(PL‐Por)	≈2 foldc)	Combined therapy led to lower tumor growth rate (105 mm^3^ day^–1^) while monotherapies (PL‐PTX 185 mm^3^/day, PDT 182 mm^3^ day^–1^, saline 206 mm^3^ day^–1^) caused no significant tumor inhibition	193
Dox and **chlorin e6** co‐loaded liposomes(PL‐Dox‐Ce6)	–	Significant more tumor inhibition (50% cure, 2 doses) vs PL‐Dox or PL‐ce6 (0% cure)	207
PoP liposomes (containing **HPPH‐lipid**) loaded with Dox	3–5 fold	Significant prolonged survival (80% cure rate, MTR over 90 days) vs empty PoP LP with light (0% cure) or Dox‐PoP without light (0% cure)	208
PoP liposomes(containing **pyro‐lipid**) loaded with Dox(Dox‐PoP liposomes)	7 fold	Significant enhanced survival (50% cure rate, 5–7 mg kg^–1^ Dox) vs empty PoP (0% cure), Dox‐PoP without light (0% cure), HPPH‐PDT(0% cure), Doxil (0% cure)	209
PoP liposomes(containing **HPPH‐lipid**) loaded with Dox	5 fold	Significantly enhanced tumor inhibition (100% cure, 10 mg kg^–1^ Dox) vs liposomal Dox (0% cure, 12.3 days of growth delay)	201
**HPPH** combined with Doxil	2–3 fold	HPPH‐PDT combined with Doxil resulted in enhanced tumor inhibition (70–80% cure, MTR >90 days) compared to PDT(18.2% cure, MTR 14 days) or Doxil (0% cure, MTR 11–15 days)	189
mAb‐conjugated **IR‐700** combined with DaunoXome	12 foldc)	Significant enhanced tumor growth inhibition with combined therapy	200

^a)^Photosensitizers used in the combined strategies are marked in bold;

^b)^MTR is defined as the median time of tumor regrowth.

^c)^Based on fluorescence imaging.

#### 4.1.1 Liposomes and Light‐Responsive Liposomes

Being able to encapsulate both hydrophobic photosensitizers and some hydrophilic drugs, liposomes are well‐suited nanocarriers for CPT. Peng et al. developed liposomes encapsulating both Dox and chlorin e6 (PL‐Dox‐Ce6) to augment the therapeutic effect of Dox‐loaded PEGylated liposomes.^207^ PL‐Dox‐Ce6 was significantly more effective than PL‐Ce6 and PL‐Dox alone or in combination. The reason that PL‐Dox‐Ce6 was more effective than the co‐injection of PL‐Ce6 and PL‐Dox may be due to the release of Dox from PL‐Dox‐Ce6 following light irradiation. The cellular distribution of free Dox, liposomal Dox and PL‐Dox‐Ce6 revealed that while PL‐Dox‐Ce6 was in the cytoplasmic area, Dox was co‐localized with nuclear staining positive signals after light irradiation, indicating photo‐induced drug release from PL‐Dox‐Ce6.

While most of the nanocarriers used for dual photodynamic therapy and chemotherapy were found to be significantly more effective than the corresponding monotherapies, how photo‐induced vasculature permeabilization leads to enhanced accumulation of nanoparticles is not fully understood in most cases. A recent study by Araki et al. demonstrated the augmented EPR effect induced by vascular PDT significantly reduced the tumor volume in a poorly permeable tumor model.^193^ The hydrophobic porphyrin derivative photoprotoporphyrin IX dimethyl ester was formulated in polymeric nanoparticles composed of PEG‐block‐polyactic acid (PN‐Por) for PDT treatment. In mice bearing C‐26 tumors with high vasculature permeability, subsequent injection of PEG liposomal paclitaxel (PL‐PTX) after PN‐Por mediated PDT treatment did not show additive effects; however, in mice bearing B16 tumors with low vasculature permeability, the therapeutic efficacy of PL‐PTX was significantly enhanced after PN‐Por mediated PDT. This study indicates that PDT can be used as a means to improve the therapeutic efficacy of nanoparticle‐based therapy in hypovascular tumors.

**Figure 5:**
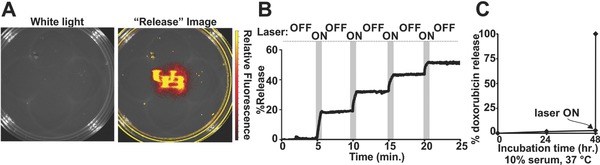
NIR light‐triggered release of cargos from PoP liposomes. A) Sulforhodamine B loaded PoP‐liposomes were stable in hot agarose (≈60 °C). When irradiated with a laser, sulforhodamine B released with high spatial control to spell “UB”. B) Transient permeabilization of calcein‐loaded PoP‐liposomes by periodic laser irradiation. C) Dox‐loaded PoP‐liposomes were stable in 10% serum for 2 days and rapidly release Dox when irradiated with 300 mW laser for 4 min. Adapted with permission.^208^ Copyright 2014, Nature Publishing Group.

The ability to release a drug, on‐demand, in a controlled and selective manner can increase the efficacy of a therapy by increasing the local concentration and its bioavailability at a specific site of interest.^17^, ^18^, ^210^ Photosensitizers, when incorporated into nanoparticles, can sometimes be used to trigger release the encapsulated contents upon NIR irradiation.^211^, ^212^, ^213^ It has been demonstrated that liposomes incorporating porphyrin‐phospholipid (PoP) can be permeabilized by NIR light, releasing variable cargos such as Dox, calcein, sulforhodamine B, and gentamicin.^208^ PoP can be used to form nanoparticles with theranostic characters.^214^, ^215^, ^216^, ^217^, ^218^ PoP lipoosmes are thermostable, as PoP liposomes loaded with sulforhodamine B did not show cargo leakage in hot agarose before solidification. However, when irradiated with laser, excellent spatial control of permeabilization and release of sulforhodamine B was observed (**Figure** **5**A). Lipid membranes can stably reseal following irradiation. As shown in Figure 5B, a NIR laser was applied intermittently to calcein‐loaded PoP liposomes. Release of calcein during laser “on” and “off” periods was assessed in real time. Calcein release only occurred during the irradiation and ceased within seconds when the laser was turned off, suggesting that PoP‐liposomes rapidly resealed and re‐formed the bilayers when irradiation was halted. Minimal release of Dox from PoP liposomes was observed when incubated in 10% fetal bovine serum for 48 h. However, when irradiated, complete release occurred in 4 min (Figure 5C).

**Figure 6:**
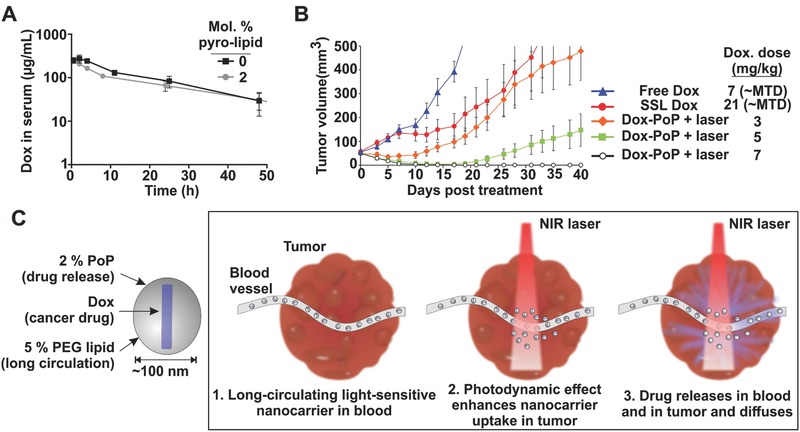
NIR light‐triggered Dox‐loaded PoP liposomes for CPT. A) Long blood circulation time of Dox loaded‐PoP liposomes. CD‐1 mice were administrated with indicated liposomes at 10 mg kg^–1^ Dox. B) Enhanced anti‐tumor efficacy of Dox‐PoP liposomes. Mia Paca‐2 xenografts were intravenously. injected with Dox‐PoP liposomes (3, 5, 7 mg kg^–1^ Dox, or 0.5, 0.9, and 1.2 mg kg^–1^ PoP), compared with sterically stabilized Dox (21 mg kg^–1^) and free Dox (7 mg kg^–1^) at maximal tolerate doses. Irradiation was initiated 1 h post injection at 250 mW cm^–2^ for 16 min 40 s (total fluence 300 J cm^–2^). C) Schematic of light‐sensitive PoP liposomes for CPT. PoP liposomes are composed of 2% Pyro‐lipid to enable light triggered release while 5% PEG‐lipid ensures long circulating property. PoP liposomes can load doxorubicin and release the drug selectively in tumors following NIR irradiation. A, B) Adapted with permission.^209^ Copyright 2015, Elsevier.

Follow‐up studies have shown that PoP liposomes containing HPPH‐lipid were not able to balance rapid light release while maintaining stability in serum.^209^ To address this, PoP liposomes comprising of Pyro‐lipid rather than HPPH‐lipid were developed.^209^ A 2 mol.% quantity of Pyro‐lipid imparted the light‐triggered capability of conventional liposomes, while 5 mol.% PEG‐lipid enable the long circulation of Dox‐loaded PoP liposomes (Dox‐PoP liposomes). Dox‐PoP liposomes demonstrated rapid light induced release in 50% serum (50% release in 1.3 min), and long circulation half‐life in mice (21.9 h, **Figure** **6**A). Following intravenous injection, Dox‐PoP liposomes were able to induce a 7‐fold increase of drug accumulation in the illuminated tumors, probably due to a combination of drug release and PDT‐induced enhanced vascular permeabilization effect. Dox‐PoP liposomes demonstrated remarkable anti‐tumor efficacy; a single treatment with 5–7 mg kg^–1^ Dox‐PoP liposomes + irradiation effectively eradicated MIA Paca‐2 tumors, and significantly enhanced efficacy compared with conventional sterically stabilized liposomal (SSL) Dox and free Dox at maximal tolerate doses (Figure 6B). Stealth Dox‐PoP liposomes with phototreatment at 3 mg kg^–1^ Dox were slightly more effective than SSL Dox at 21 mg kg^–1^. A combination of enhanced drug accumulation, drug release and photodynamic therapy are likely to contribute to the superior efficacy of this treatment. Figure 6C is a schematic of using PoP liposomes for CPT. Following intravenous injection of long circulating, light sensitive liposomes, a NIR laser is applied to tumors to initiate phototreatment. Through PDT effects, tumor blood vessels are permeabilized for enhanced tumor uptake of nanoparticles, and the laser treatment also induces the release of the encapsulated Dox from the liposomes.

The effects of metal chelation (copper and zinc) to PoP can modulate the phototherapeutic properties of PoP liposomes.^219^ It was found that Cu (II) and Zn (II) PoP liposomes containing 10 mol.% HPPH‐lipid, demonstrated unique photophysical properties and released cargo in response to NIR light. Cu‐PoP liposomes exhibited minimal fluorescence and reduced production of reactive oxygen species upon irradiation. Zn‐PoP liposomes retained fluorescence and singlet oxygen generation properties; however, they rapidly self‐bleached under irradiation. Compared to the free base form of HPPH, both Cu (II) and Zn (II) PoP showed reduced phototoxicity in mice.

**Figure 7:**
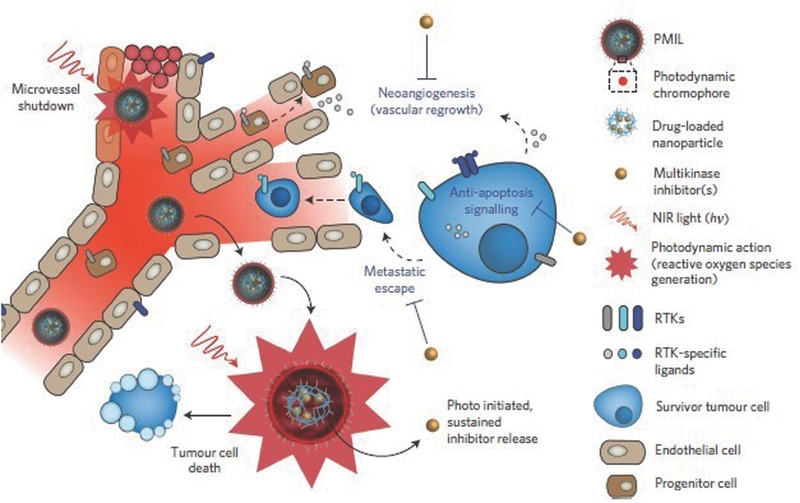
PMIL delivery system for CPT. NIR light initiates a PDT response, including tumor cell apoptosis and necrosis, neovascular damage, as well as disruption of liposomes and sustained release of cabozantinib which inhibit both MET and VEGF signaling pathways, resulting in the further suppression of tumor cell survival, metastasis and regrowth. Reproduced with permission.^220^ Copyright 2016, Nature Publishing Group.

Another NIR particle, light‐activated multi‐inhibitor nanoliposome (PMIL), was loaded with cabozantinib, a multikinase inhibitor developed for CPT.^220^ As demonstrated in **Figure** **7**, PMIL consists of a liposome with a photoactivable chromophore (benzoporphyrin derivative, BPD) in the lipid bilayer and a nanoparticle containing cabozantinib encapsulated. Light irradiation followed by intravenous PMIL administration led to PDT damage of tumor cells and microvessels, and release of cabozantinib in the tumor. Cabozantinib can simultaneously inhibit VEGF and MET pathways. MET signaling promotes escape from cytotoxic and antiangiogenic therapy via supporting cancer cell survival, motility and metastasis. A single PMIL treatment allowed for prolonged tumor reduction in two mouse models and suppression of metastatic escape in an orthotopic pancreatic tumor model.

#### 4.1.2 Polymeric Micelles

**Figure 8:**
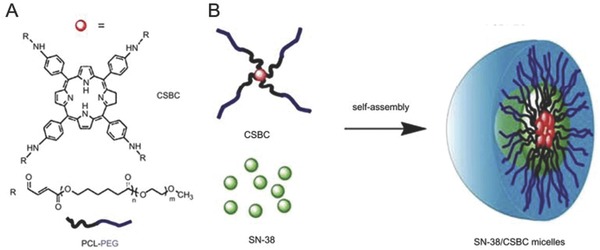
A) Structure of a chlorin‐core star shaped diblock copolymer (CSBC) and B) Schematic of SN‐38 loaded self‐assembled CSBC micelles. Reproduced with permission.^204^ Copyright 2009 Elsevier.

Polymeric micelles have also been used as a dual carrier for CPT. Peng et al. developed chlorin‐core star shaped diblock copolymer (CSBC) micelles for chemo‐photodynamic therapy.^204^ The structure of a CSBC micelle monomer is shown in **Figure** **8**A. The hydrophobic chemotherapeutic agent paclitaxel was encapsulated inside the diblock micelles. In vitro studies demonstrated that these chlorin core micelles significantly increased the cytotoxicity of paclitaxel in MCF‐7 cells through a synergistic effect. In a follow up study, CSBC micelles were loaded with another hydrophobic drug, SN‐38, a biologically active form of irinotecan hydrochloride (CPT‐11).^204^ The SN‐38/CSBC micelle, the structure of which is illustrated in Figure 8B, increased the blood circulation time and tumoral drug accumulation compared to the free form of the drug. SN‐38 loaded CSBC mediated PDT synergistically inhibited tumor growth, with 3 doses of SN‐38/CSBC and 3 light treatment (total light dose 90 J cm^–2^) and resulted in 60% complete tumor regression (defined as no palpable tumor on day 30). Tumor microvessel density and cell proliferation treated with micellular SN‐38/CSBC, with or without PDT were significantly decreased, which is in accordance with the anti‐angiogenesis effects of CPT‐11.^221^ The anti‐angiogenesis effect from CPT‐11 could led to the enhancement of the combinatorial treatment outcome.

#### 4.1.3 Polymer‐Drug Conjugates

Hydrophilic polymers such as N‐(2‐hydroxypropyl)methacrylamide (HPMA), PEG, and polyglutamate (PGA) are frequently used to make polymer‐drug conjugates, and have similarly been used to deliver PDT agents.

By conjugating meso‐chlorin e_6_ monoethylene diamine disodium (Mce_6_) or Dox to HPMA, Peterson et al. demonstrated a significant improvement in tumor cures for the combination of chemotherapy (HPMA‐adriamycin conjugates, P‐A) and PDT (HPMA‐Mce_6_ conjugate, P‐C) compared to P‐A or P‐C with light alone in human ovarian carcinoma xenografts.^202^ Notably, P‐A, demonstrated a greater safety margin than Dox. Similarly, the water soluble polymer HPMA conjugated Mce_6_ resulted in reduced nonspecific toxicity. The HPMA copolymer‐conjugated drugs demonstrated long circulation times and in particular, significantly higher levels of drugs in the tumor tissues compared to the free drugs due to the EPR effect.^222^ Combination of P‐A (2.2 mg kg^–1^ Dox equivalent) and P‐C (8.7 mg kg^–1^ Mce_6_ equivalent) with light was safe and resulted in significant improvement in tumor cure than P‐A alone or P‐C with light. Multiple light treatments can be performed to increase the anti‐tumor efficacy of the combination therapy.^203^ The anti‐tumor effectiveness was ranked as multiple PDT plus multiple P‐A was more effective than multiple PDT, which was more effective than single PDT plus multiple P‐A, which was more effective than multiple P‐A.

The same group developed Fab' fragment targeted HPMA copolymer‐drug conjugates and evaluated these against ovarian carcinoma OVCAR cells.^223^ The anti‐cancer drugs SOS thiophene was covalently conjugated to the targeted HPMA and used concurrently with non‐targeted copolymer‐Mce_6_ for CPT. In vitro results confirmed enhanced binding and internalization in OVCAR cells.

#### 4.1.4 Other Nanoparticles

Development of drug resistance due to overexpression of efflux transporters such as P‐glycoprotein (P‐gp) can limit the clinical therapeutic outcomes of chemotherapy.^224^ Photosensitizers such as methylene blue may be able to inhibit P‐gp mediated drug efflux and this was independent of PDT effect, as methylene blue without light can counter the drug resistance of Dox in cell lines.^225^ Nanoparticles combining two treatment modalities using Dox and methylene blue can potentially overcome tumor drug resistance. Dox and methylene blue were designed to release upon illumination following endocytosis of nanoparticles, while methylene blue could potentially reverse multiple drug resistance (MDR). Khdair et al. demonstrated that Aerosol OT (AOT)‐alginate nanoparticles can be used as a carrier for simultaneous delivery of Dox and methylene blue.^205^, ^226^ The enhancement of efficacy of combining PDT and chemotherapy was demonstrated in drug‐resistant NCI/DOX‐RES cells.^226^ The improvement in cytotoxicity could be due to the improved intracellular and nuclear delivery of both drugs. The same group later evaluated the efficacy of these nanoparticles loaded with methylene blue and Dox in the drug resistant JC tumor model.^205^ Nanoparticle‐mediated combinatorial treatment significantly inhibited tumor growth and improved survival. It was demonstrated that the combinatorial therapy resulted in a significant induction of apoptosis and necrosis and tumor microvasculature damage.

**Figure 9:**
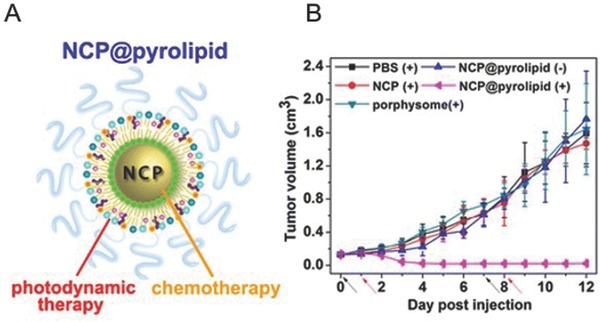
Core‐shell nanoparticles for CPT of resistant head and neck cancers. A)Schematic of self‐assembled NCP@pyrolipid core–shell nanoparticle with pyrolipid and PEG‐lipid in the outlayer and platinum‐based prodrugs in the core. B) In vivo anti‐tumor efficacy of NCP@pyrolipid. PBS, NCP, porphysome, or NCP@pyrolipid were intravenously injected into SQ20B subcutaneous xenograft mouse models at a cisplatin dose of 0.5 mg kg^–1^ or pyrolipid dose of 0.5 mg kg^–1^ followed by irradiation (100 mW cm^–2^ for 30 min, total 180 J cm^–2^) 24 h post injection. Reproduced with permission.^206^ Copyright 2015, American Chemical Society.

He et al. developed nanoscale coordination polymer (NCP)‐based core‐shell nanoparticles (NCP@pyrolipid) carrying a high amount of cisplatin and the photosensitizer pyro‐lipid, for combined chemo‐photodynamic therapy.^206^ The schematic of NCP@pyrolipid is shown in **Figure** **9**A. The NCP core was developed by linking platinum‐based prodrugs with zinc metal ions via coordination bonds and then coated with lipid bilayer.^227^ Synergy with chemotherapy from cisplatin and PDT was observed in head and neck cancer cell lines and a cisplatin‐resistant SQ20B human head and neck cancer xenograft mouse model. As shown in Figure 9B, only NCP@pyrolipid+irradiation showed significant tumor regression, with a reduction of tumor volume by ≈83%. Mice in the other four groups shared similar tumor growth rates which suggest that chemotherapy or PDT alone was ineffective in inhibiting tumor growth in this model. Pharmacokinetic and biodistribution studies demonstrated prolonged circulation time, high tumor uptake and reduced distribution of cisplatin and pyro‐lipid in healthy organs.

There have been several other studies involving the co‐delivery of photosensitizers and anticancer drugs for cancer treatments. Chang et al. developed Dox‐loaded hematoporphyrin (HP)‐modified bovine serum albumin nanoparticles (HP‐NPs).^228^ In vitro studies and in vivo anticancer efficacy demonstrated the enhanced the cytotoxicity by Dox‐loaded HP‐NPs compared with HP. Using graphene oxide (GO) as a drug delivery system, Zhou et al. co‐loaded hypocrellin A (HA) and 7‐ethyl‐10‐hydroxycamptothecin (SN‐38) through noncovalent π–π stacking and hydrogen bond with GO.^229^ HA/SN‐38/GO‐mediated combination therapy exhibited synergistic cytotoxicity effect compared with PDT and chemotherapy alone.

### 4.2 Chemo‐Photothermal Systems

Besides PDT, photothermal therapy (PTT) has also shown to be able to enhance local drug delivery. Light‐absorbing photothermal agents can serve as heat carrier for local hyperthermia. Like PDT, mild hyperthermia improves vascular perfusion and enhances the extravasation of nanoparticles into the tumor interstitial space.^230^, ^231^, ^232^ Kong et al. demonstrated that liposomal extravasation rate is affected by particle size, temperature and timing between hyperthermia and liposome administration.^231^ The same work also showed that tumors that were heated could achieve a 2–4‐fold increase of liposome uptake. The combination of photothermal therapy and chemotherapy have shown synergistic effect in tumor control.^233^, ^234^ There have been research efforts in combining NIR photothermal therapy and chemotherapy to enhance the cytotoxicity of chemotherapeutic agents. Gold nanomaterials, carbon nanotubes and indocyanine green (ICG) are photothermal agents of interest due to their absorption in the NIR window.

#### 4.2.1 Gold Nanoparticles

Gold nanoparticles have been the focus of a significant amount of biomedical research in recent years. These nanoparticles absorb light in the visible region due to surface plasmon resonance (SPR) oscillations.^235^ The absorbed NIR light can be converted into heat and used as photothermal energy and can also be used to trigger cargo release.

Gold nanoparticles can be modified into different forms, such as gold nanorods (GNRs), nanoshells, nanospheres, and nanocages. The size and shape of the gold nanoparticles affects the SPR frequency.^235^ GNRs are attractive as their absorption spectrum is very sensitive to the aspect ratio (length/width) and can be fine‐tuned to absorb within the NIR region. For gold nanoshells, their optical resonance can be tuned by adjusting the ratio of the thickness of the gold shell to the diameter of the silica core.

**Figure 10:**
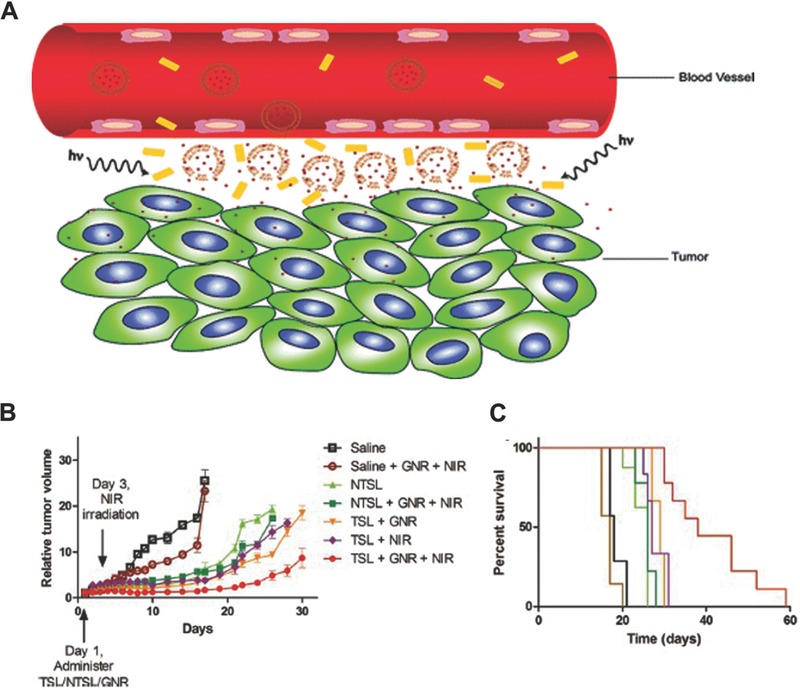
Phototherapeutic efficacy of thermosensitive liposomes with gold nanorods. A) Schematic of synergistic application of thermosensitive liposomes and gold nanorods for remote triggered drug release. B) Relative tumor volume after treatment of different groups. Mice received either liposomal Dox (2.5 mg ml^–1^) or saline in combination with GNRs; 48 h later, NIR irradiation was applied for 10min. C) Percent survival for different treatment groups. TSL + GNR + TSK was significantly different from all other groups. Reproduced with permission.^237^ Copyright 2011 American Chemical Society.

Using GNRs as a carrier to deliver heat, Agarwai et al. developed a way to remote trigger the release of Dox from thermosensitive liposomes to increase the bioavailability of liposomal Dox (**Figure** **10**A).^236^ The composition of thermosensitive liposomes was optimized to balance the serum stability and heat‐triggered release capability. To maintain stability in serum, 30% cholesterol was included in the thermosensitive liposomes (TSLs), and 3% of DMPC was incorporated to enable the heat‐triggered release. A U87‐MG xenograft mouse model was used to assess the therapeutic efficacy of synergistic application of TSLs and GNRs, compared to non‐thermosensitive liposomes (NTSLs) and GNRs alone. Liposomal drug and GNRs were co‐injected and NIR was applied 48 h later. It was demonstrated that GNR‐mediated heating of TSL was effective in suppressing tumor progression, while mice receiving NTSL + GNR + NIR were less successful (Figure 10B,C). The increased animal survival proved that GNR‐mediated heating resulted in increased bioavailability of Dox encapsulated in TSLs.

Lei et al. developed thermosensitive liposomes based on a copper–Dox complex that were responsive to both pH and heat.^237^ Synergistic application of GNRs and dual pH/thermal sensitive liposomes with NIR light produced a precise and localized temperature which remotely controlled Dox release and efficiently inhibited tumor growth.

In addition to being used as a photothermal agent, GNRs have also been designed for simultaneous delivery of heating and chemotherapeutics. Shen et al. developed mesoporous silica‐encapsulated gold nanorods (GNRs@mSiO_2_) as a synergistic therapy for delivery of heat and drugs to tumors.^238^ Dox was loaded into GNRs@mSiO_2_ which can be released upon light irradiation. In vivo studies demonstrated that compared to chemotherapy or photothermal treatment alone, the combined treatment exhibited a synergistic effect, leading to more effective therapeutic efficacy. You et al. developed Dox‐loaded hollow gold nanospheres as a platform for simultaneous photothermal cell killing and drug release.^239^ Dox was loaded into hollow gold nanospheres (HAuNS) coated with polyethylene glycol (PEG). Lee et al. also developed Dox loaded hollow gold nanoshells for photothermal ablation and chemotherapy.^240^ They also demonstrated that fluorescence optical imaging and photoacoustic imaging are promising approaches to map Dox release and monitor temperature.

**Figure 11:**
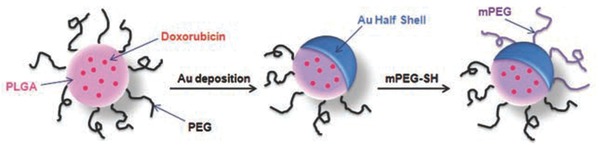
Multifunction nanoparticles comprising Dox‐loaded PLGA‐Au H‐S. Dox is encapsulated within the biodegradable PLGA NPs, and an Au layer is deposited on these NPs. Au is functionalized with thiol‐terminated methoxy‐PEG to improve the stability of the nanoparticles in physiological condition. Heat can be generated locally upon NIR irradiation and Dox can be released through the open half of the shell. Reproduced with permission.^242^

Park et al. developed multifunction nanoparticles for a combined Dox and photothermal treatment.^241^, ^242^, ^243^ As shown in **Figure** **11**, these multifunction nanoparticles were comprised of poly (lactic‐*co*‐glycolic acid) (PLGA) and loaded with Dox, with a gold layer deposited on these nanoparticles (Dox‐loaded PLGA‐Au H‐S NPs). Heat could be generated locally upon NIR irradiation. Dox release was dependent on the biodegradation of the nanoparticles and release occurred more rapidly upon irradiation due to the elevated temperature. Compared with chemotherapy or photothermal treatment alone, the combined treatment demonstrated a synergistic effect, resulting in more efficient tumor control.

#### 4.2.2 Nanographene Oxide

Nanographene oxide (NGO) has been used in drug delivery and photothermal therapy. NGO has efficient loading capacity and superior photothermal sensitivity.^244^ Zhang et al. proved the synergistic effect of chemo‐photothermal therapy using Dox loaded PEGylated graphene oxide(NGO‐PEG).^234^ Dox was loaded onto NGO‐PEG by simply mixing Dox solutions under controlled pH whereas Dox release is pH‐dependent. When administered, EMT6 tumor bearing mice that were treated with combined treatment demonstrated a synergistic effect and higher therapeutic efficacy compared to those received chemotherapy or photothermal therapy alone. Using graphene oxide as nanocarrier, Tran et al. developed a platform for dual‐drug CPT to overcome drug resistance in cancer.^245^ Dox and irinotecan (IRT) were co‐loaded into GO (DI‐GO). Following exposure to NIR light, a synergistic effect and higher efficacy to resistant breast cancer cells MDA‐MB‐231 were observed compared to chemotherapy or photothermal treatment alone. Li et al. engineered a Pluronic F127 functionalized magnetite/graphene nanohybrid (GN/Fe_3_O_4_/PF127) for CPT.^246^ F127 functionalization allowed for dispersity and stability of the nanohybrid while Fe_3_O_4_ modification imparted MRI contrast for diagnosis purpose. Dox was further loaded into this nanohybrid, and demonstrated significant cytotoxicity to HeLa cells when subjected to irradiation.

#### 4.2.3 Polymeric Nanoparticles

**Figure 12:**
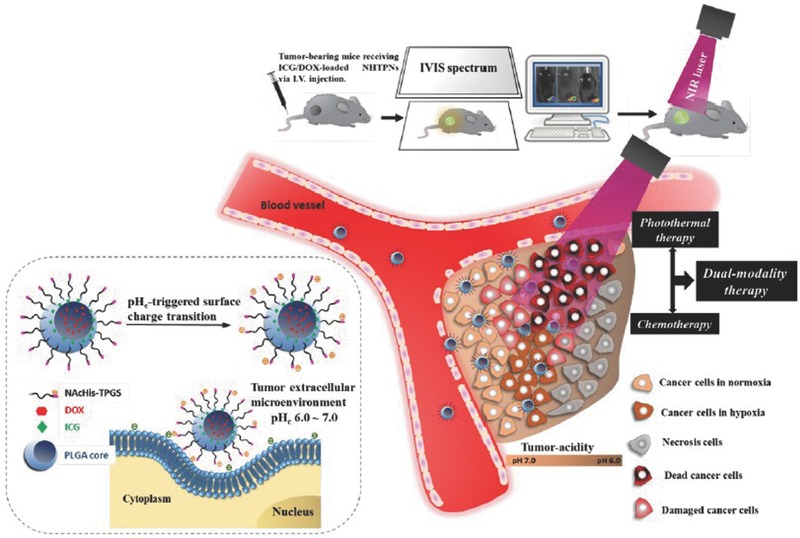
Active tumor penetration and uptake of ICG/Dox loaded nanoparticles with pH‐triggered surface charge transition for image‐guided chemo‐photothermal therapy. Reproduced with permission.^247^ Copyright 2016 Ivyspring.

Hung et al. developed a pH‐responsive surface charge‐switchable nanoparticles co‐loaded with a photothermal agent, indocyanine green (ICG) and Dox.^247^ As shown in **Figure** **12**, This nanovehicle system is composed of poly(lactic‐co‐glycolic acid) (PLGA) which acts as the hydrophobic cores, and coated with pH‐responsive N‐acetyl histidine modified D‐α‐tocopheryl polyethylene glycol succinate (NAcHis‐TPGS). The nanocarriers are sensitive to the tumor extracellular acidity. The in vitro cellular uptake of ICG/Dox‐loaded nanoparticles by cancer cells and macrophages was significantly improved in weak acidic environment due to the increased protonation of the NAcHis residues. It was demonstrated that these nanoparticles can substantially accumulate in solid tumors and active permeation of the nanoparticles into deep tumor hypoxia areas were confirmed. These nanoparticles demonstrated significant efficacy in suppressing tumor growth and could be used for image‐guided photothermal therapy due to the high fluorescence quantum yield of ICG.

**Figure 13:**
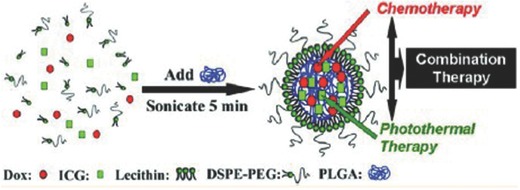
Single‐step assembly of Dox/ICG loaded lipid‐polymer nanoparticles (DNIPs) for CPT. DINP comprises a hydrophobic PLGA core, a hydrophilic PEG shell, and a lipid (lecithin) monolayer at the interface of the core and shell with Dox and ICG encapsulated. Reproduced with permission.^249^ Copyright 2013, American Chemical Society.

Self‐assembled lipid‐polymer hybrid nanoparticles have been demonstrated to be a robust drug delivery platform.^248^, ^249^, ^250^ These nanoparticles comprise a hydrophobic PLGA core, a hydrophilic PEG shell, and a lipid (lecithin) monolayer at the interface of the core and shell that acts as a molecular fence to enhance drug retention.^248^ Using a single‐step sonication method, Zheng et al. developed Dox/ICG loaded lipid‐polymer nanoparticles (DINPs) for chemo‐photothermal therapy (**Figure** **13**).^249^ The DINPs demonstrated a higher temperature response and faster Dox release under laser irradiation. The cytotoxic effects of DINPs was assessed in xenografts of Dox‐sensitive MCF‐7 tumors and Dox‐resistant MCF‐7/ADR tumors. In comparison with chemotherapy or photothermal therapy alone, a single dose of DINPs with laser irradiation synergistically suppressed tumor growth and no recurrence was seen.

Hao et al. developed multifunctional PLGA nanoparticles for simultaneous delivery of ICG and docetaxel (DTX) to the brain through surface decoration with the brain‐targeting peptide angiopep‐2 (ANG/PLGA/DTX/ICG NPs).^251^ ICG is used as a NIR imaging and photothermal agent while DTX is a chemotherapeutic agent. NIR image‐guided CPT of the ANG/PLGA/DTX/ICG NPs induced U87MG cell death in vitro and significantly prolonged the survival time of the mice bearing orthotopic U87MG glioma xenografts. Wang et al. also designed multifunctional micelles for multimodal imaging and CPT of the drug‐resistant tumor.^252^ These micelles comprised of pH‐sensitive diblock copolymer PEG‐b‐PDPA, gadolinium‐coordinated photosensitizer Ce6, and a pluronic prodrug Dox. These micelles were designed for multimodal imaging including fluorescence imaging, MRI and photoacoustic imaging and combinational therapy including PDT, PTT and chemotherapy.

### 4.3 Other CPT systems

**Figure 14:**
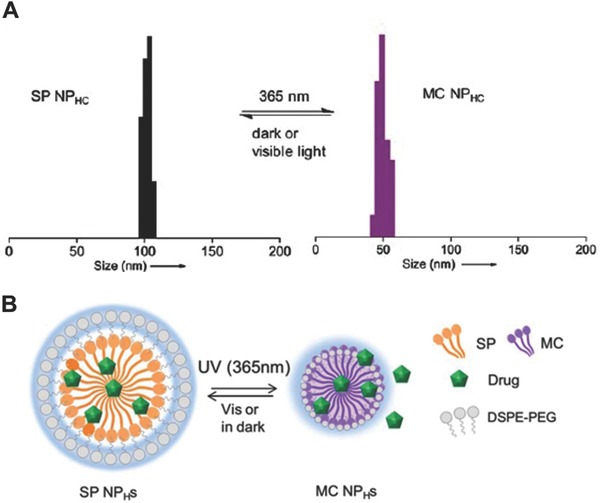
Photoswitchable nanoparticles for triggered tissue penetration. A) Size changes upon alternating UV (35 s) and visible light illumination (500–600 nm, 5 min, 0.5 W cm^–2^). B) Scheme of photoswitchable SP/DSPE‐PEG lipid hybrid nanoparticles (NP_H_s). Reproduced with permission.^253^ Copyright 2013, National Academy of Sciences.

Other CPT systems have been described that do not rely on PDT or PTT. Tong et al. developed spiropyran‐based nanoparticles which use UV light to remotely trigger a reversible change in particle volume.^253^ The photoswitching nanoparticles were comprised of spiropyran (SP) and lipid‐polyethylene glycol (PEG). Upon irradiation at 365 nm, these nanoparticles shrank from 103 nm to 49 nm (**Figure** **14**A)^253^ due to the switch of hydrophobic SP to zwitterionic merocyanine (MC) upon irradiation which alters the physical assembly properties (Figure 14B).^254^ The shrinkage leads to enhanced penetration into tumors and drug release. Such nanoparticles loaded with docetaxel were more effective than free docetaxel or encapsulated docetaxel without irradiation. The enhanced efficacy of nanoparticles in irradiated tumors may be related to the enhanced penetration by nanoparticles and decompression of tumor blood vessels.

Many light controlled release nanoparticle systems suffer from a common problem of cargo leakage upon administration, before the application of the trigger. Covalent conjugation of the anti‐cancer drugs to the carrier is one strategy for developing more stably loaded nanocarriers. Wong et al. recently reported upconversion nanocrystals (UCNs), with the drug, Dox, covalently conjugated to the carrier via a photocleavable linker.^255^ This core–shell‐based system has NIR photoluminescence and can potentially be used for NIR imaging and drug delivery. When excited by NIR (980 nm), the UCNs can convert the absorbed NIR to shorter visible and UV light which can be harnessed to trigger the release the conjugated payload through a photochemical mechanism. Folate‐conjugated dendrimers were also integrated to the UCNs for tumor targeted delivery of Dox. The selective binding and uptake of these UCNs were verified in vitro.

## 5 Clinical Outlook and Conclusions

Phototherapies, including PDT and laser‐induced thermal therapy, have proven to be effective treatments for certain cancers. However, PDT has been slow in becoming a mainstream cancer therapy for solid tumors, possibly due to treatment complexity or a lack of large scale of clinical trials validating the benefits of PDT.^58^, ^76^ It has also been challenging to establish optimum treatment parameters such as the drug and light dose, and the drug‐to‐light interval.^256^ CPT may present further complications with respect to dosing parameters. Chemotherapy is a treatment option for many cancer patients. However, this single modality is often not curative, and is limited by the dose‐limiting systemic toxicity and drug resistance. These drawbacks lead to search of more effective and safer cancer therapy or mechanism‐based combination therapies that overcome these limitations.

A large number of preclinical and clinical works have demonstrated that the combination of PDT with conventional chemotherapeutics (e.g., Dox, cisplatin) are more effective than the monotherapies. The mechanisms of these synergistic effect are not only a direct sum of damages caused by both modalities, but also due to effects on the tumor vasculature and in some cases, induction of an immune response which helps fight against the cancer cells. However, the rationale and understanding of treatment procedure such as whether chemotherapeutics should be administered before or after irradiation are still not well understood. More detailed studies on the mechanisms need to done to understand the cooperation of chemotherapy and PDT on a molecular basis. Combinations of PDT with novel anti‐cancer agents (e.g., COX‐2 inhibitors, anti‐angiogenesis agents) that nullify the PDT‐triggered molecular responses such as the increase of angiogenenic and survival molecules have also been proved to be promising strategies.

Nanoparticle‐based CPT offers extra mechanisms of enhanced efficacy, in particularly the photo‐induced enhanced vasculature permeabilization effect, which leads to substantial increase in drug accumulation in the irradiated tumor. Nanoparticle CPT also possibly represents a simpler paradigm in which a single agent (with dual function) is administered to patients. The concept of PDT‐induced vasculature permeabilization to allow for better delivery of nanotherapeutics should be studied in greater detail. Treatment parameters such as drug dose, photosensitizer and light dose, and drug–light interval need to be studied in detail to understand and optimize this effect for better anti‐tumor outcomes. As nanoparticles for CPT contain two active components (chemotherapeutics and photosensitizers), balancing the dosimetry of each component is more challenging than that of the nanoparticles for monotherapies and there are also material‐side constraints imposed by integrating the components into a single agent. In addition to the laser‐induced enhanced drug accumulation, nanoparticle‐based CPT can offer triggered drug release capability which can potentially increase the bioavailability of nanomedicines and improve efficacy. However, the impact of light‐triggered release in CPT has not yet been determined and needs to be better assessed. The speed of the light‐triggered release may be important and there have been recent efforts focused on accelerating release speed.^257^ Nanoparticles that combine chemotherapy and phototherapy into one agent have demonstrated favorable anti‐tumor responses.

As the synergistic efficacy of CPT derives from both chemotherapy and phototherapy, the potential limitations of CPT are also related to these two modalities. With regard to PDT, photosensitivity prevents patients from sunlight exposure for a certain amount of time, depending on the type of photosensitizer used. As light has a short penetration depth, treatment procedures for deep‐seated or large tumors may be complex, and may involve the intersttial insertion of multiple optical fibers and accurate determination of the ablation zone. Owing to the chemotherapy component, CPT may induce typical chemotherapy side effects such as immunosuppression. However, since CPT should enable the use of lower dose of chemotherapeutics, side effects in this regard should be reduced. CPT represents a relatively unexplored and potent tumor treatment modality that warrants further research in larger animals and in clinical studies.
